# A Quantum Chemical Topology Picture of Intermolecular Electrostatic Interactions and Charge Penetration Energy

**DOI:** 10.1021/acs.jctc.1c00263

**Published:** 2021-07-19

**Authors:** Fernando Jiménez-Grávalos, Dimas Suárez

**Affiliations:** Departamento de Química Física y Analítica, Universidad de Oviedo, E-33006 Oviedo, Spain

## Abstract

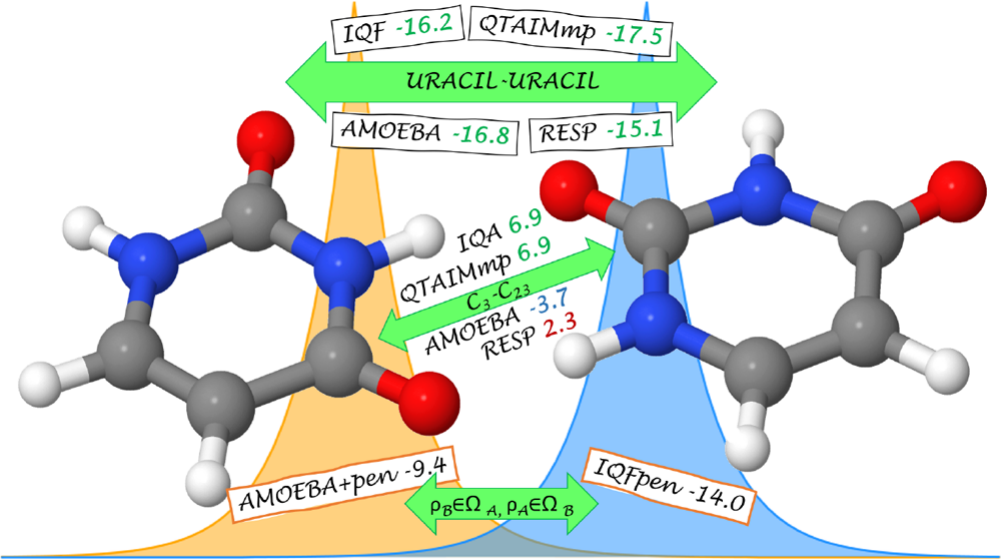

Based on the Interacting Quantum Atoms approach, we present herein a conceptual and theoretical framework of short-range electrostatic interactions, whose accurate description is still a challenging problem in molecular modeling. For all the noncovalent complexes in the S66 database, the fragment-based and atomic decomposition of the electrostatic binding energies is performed using both the charge density of the dimers and the unrelaxed densities of the monomers. This energy decomposition together with dispersion corrections gives rise to a pairwise approximation to the total binding energy. It also provides energetic descriptors at varying distance that directly address the atomic and molecular electrostatic interactions as described by point-charge or multipole-based potentials. Additionally, we propose a consistent definition of the charge penetration energy within quantum chemical topology, which is mainly characterized in terms of the intramolecular electrostatic energy. Finally, we discuss some practical implications of our results for the design and validation of electrostatic potentials.

## 1 Introduction

Electrostatic interactions are central to molecular modeling because of their slow decay and strength. Especially when polar atoms or charged species are involved, they largely determine the stability and activity of biomolecules such as proteins, nucleic acids, or lipids, among others.^1,2^ As such, a reliable description in molecular mechanics (MM) potentials is essential both in the short- and in the long-range.

Within the framework of MM methods, interactions comprising nonbonded atoms are usually represented by pairwise potentials such as the Lennard-Jones and the Coulomb ones. In the latter case, the use of point charges or higher order multipoles to avoid the integration of interacting charge densities has resulted in accurate electrostatics at long-range, with significant improvements to speed up and facilitate convergence such as the Ewald summation and its variants to perform, for example, molecular simulations in solution under periodic boundary conditions.^3−8^ At short-range, however, the approximations taken for long distances become less accurate or invalid,^9^ and a correct electrostatic description in this regime stills poses a challenge. Hence, there is a growing interest in improving short-range electrostatics (e.g., for troublesome hydrogen bonds), mainly focused on capturing the effects associated with the non-negligible interpenetration of densities, leading to the so-called charge penetration (CP) energy, which is typically defined as the difference between the electrostatic energy computed from continuous charge density distributions and that from multipolar approximations.^10^ Thus, several investigations have been devoted in the last years to incorporate the charge penetration energy into the MM electrostatic potentials.^10−14^

The separation of various energy terms as implemented in the MM potentials is somehow paralleled by the energy decomposition analysis (EDA) methods.^15^ A major goal of any EDA approach is to ascertain the nature and type of the interactions among molecules as well as to rationalize their stabilizing or destabilizing roles, which may have implications for the design, parametrization, and validation of MM potentials such as the electrostatic ones. However, there is no unique recipe to decompose the energy, and thus many EDAs have been developed rooted in different approaches. Hence, symmetry-adapted perturbation theory (SAPT) makes use of a perturbative approach to differentiate the distinct nature of the intermolecular interactions,^16,17^ while orbital-based EDAs exploit a stepped scheme to calculate the different energies according to some reference electronic states,^18−20^ and the interacting quantum atoms (IQA) method relies on a real space partition of the quantum mechanical (QM) density matrices,^21,22^ being thus classified as a quantum chemical topology (QCT) method.

According to recent studies, in spite of their crude approximations, it may be feasible to improve the classical MM potentials by utilizing the information provided by EDAs.^23^ More specifically, it has been shown that the SAPT energy components (electrostatics, induction, exchange-repulsion, and dispersion) can be modeled with relatively simple MM functions.^24,25^ In particular, it has been demonstrated that the combination of empirical damping functions accounting for the CP energy with point multipoles results in electrostatic energies at short-range that are quite close to the SAPT ones. Actually, the SAPT electrostatic energy provides the required reference to parametrize and validate the CP-augmented potentials. However, different interpretations of short-range energetic effects involving the overlap of the electron densities of two or more fragments may be possible depending on the particular EDA of choice.^15^ As such, other schemes such as the absolutely localized molecular orbital (ALMO) EDA, that relies on a different nonperturbative decomposition of energy terms, have also been proposed.^26^ In this work, we reexamine the nature of electrostatic interactions under the prism of an orbital-invariant, reference-free technique. The IQA approach fulfills these requirements as it is a QCT, real-space energy decomposition resorting to the partition of the reduced density matrices (RDMs). IQA distinguishes not only between electrostatic or exchange-correlation components of the interaction energy but also between intra- or interatomic (or fragment) contributions. Moreover, since IQA splits the total energy of a system and not only the interaction between selected fragments, it is capable of reconstructing (or dissecting) the energy ascribed to both covalent and noncovalent binding, allowing thus covalent bond energies to be characterized^27^ as well as the accuracy of the energy components handled by QM fragment methods to be investigated.^28^

Herein, we study in detail the electrostatic interactions involved in noncovalent complexes with a twofold goal. On the one hand, we aim to compare in a consistent and systematic manner the atomic and fragment contributions to the electrostatic energy as evaluated throughout a hierarchy of QM and MM approximations and at varying intermolecular distances. In this way, we seek to identify the best correspondence between the IQA and the MM electrostatic terms. On the other hand, we critically examine the CP concept and propose a novel definition relying on a joint orbital and real-space decomposition scheme, which can give new insight into the CP energy. To help fulfill these goals, the rest of the manuscript is structured as follows. First, we present and describe the theoretical scaffold that holds our work, paying particular attention to the IQA—and its IQF variant—energy decomposition, followed by subsections concerning the zeroth-order approximation, the electrostatic MM potentials, and a final assessment of the CP energy and the alternative definition proposed in this work. Subsequently, we describe some computational settings and the results of our test calculations, which were carried out on the S66 and S66x8 data sets.^29,30^ The various levels of description of the electrostatic interactions are then discussed based on their statistical correlation with benchmark data, their dependence with the intermolecular separation, etc. The QM and IQA calculations yield further information, not only about the magnitude of the CP energy, but more importantly, about its different role in the IQA descriptors. Finally, we conclude that the aim of improving the electrostatic description is essentially fulfilled at the expense of accounting for intramolecular effects.

## 2 Theory and Methods

### 2.1 IQA Decomposition of QM Energies

The interacting quantum atoms method is a robust and physically sound approach to decompose the total QM energy of a system into chemically meaningful components.^21,22^ It is based on partitioning the first- and second-order RDMs, as can be done with the real space partition proposed by Bader and co-workers within their Quantum Theory of Atoms in Molecules (QTAIM).^31^ Thus, the three-dimensional space is decomposed into atomic regions (Ω_*I*_) as the attraction basins of the gradient field of the electron density.

Given a global energy *E* of a system, IQA permits its decomposition into atomic components and pair interaction energies according to
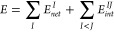
1where  is called the net atomic energy and, under the Born–Oppenheimer approximation, represents the energy due to the kinetic energy of electrons plus all the interactions involved (i.e., electron–electron and electron–nucleus) inside the atomic basin of each atom *I*. Similarly, each  term comprises the interaction energy between the electrons (e) and nucleus (n) located in atom *I* with those ascribed to other atoms *J*, which can be separated into n–e, e–e, and n–n contributions.

In order to compute the potential energy, the pair density ρ_2_(**r**_1_, **r**_2_) is required. This object can be split according to ρ_2_(**r**_1_, **r**_2_) = ρ(**r**_1_)ρ(**r**_2_) + ρ_*xc*_(**r**_1_, **r**_2_) in two contributions. On the one hand, ρ(**r**_1_)ρ(**r**_2_) represents a noncorrelated product of densities, whereas electron correlation is accounted for by the exchange-correlation (xc) density ρ_*xc*_(**r**_1_, **r**_2_). Accordingly, the total interaction energy between two atoms can be decomposed into a Coulomb or electrostatic term  and a quantum mechanical exchange-correlation one :
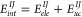
2the latter term comprising all the associated QM effects that other (e.g., perturbative) approaches identify separately as dispersion, charge-transfer, polarization, etc. However, such a decomposition of  into two terms is particularly relevant when assessing the nature of a given bond or interaction, since the electrostatic term is associated with ionicity and the exchange-correlation contribution with covalency.^22^

IQA admits the grouping of atomic terms into fragment contributions (e.g., functional groups, molecules). Thus, a fragment decomposition similar to eq 1 of a molecular aggregate constituted by two moieties *A* and *B* involves
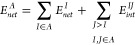
3
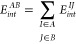
4where  can be calculated analogously. For practical purposes, we use the IQA acronym to refer to the atomic analysis, whereas for its fragment version the term interacting quantum fragments (IQF) is preferred.

In a previous work,^32^ it was shown that IQF may be useful to dissect the formation energy of noncovalent complexes. Moreover, the IQA/IQF terms can be augmented with Grimme’s D3 dispersion correction^33^ as combined with the Becke-Johnson damping function^34^ to complement the DFT and HF descriptions. Using the IQF-D3 protocol, the formation energy of a two-fragment system *AB* given by the process *A* + *B* → *A*···*B* is split as
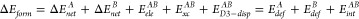
5The deformation term  () in the above equation corresponds to the net energy variation  () of fragment *A* (*B*), whereas the interfragment interaction energy  comprises the electrostatic (), exchange-correlation ), and dispersion () energies between the two fragments, the latter being thus separated from the whole exchange-correlation one. Overall, the contribution of electrostatics and exchange-correlation to Δ*E*_*form*_ is split between the intrafragment deformation and the interfragment interactions.

### 2.2 Electrostatic Energy from Continuous Charge Densities

The purely electrostatic energy for a given system with total charge density ρ(**r**) (ρ(**r**) ≡ ρ_*tot*_(**r**) = , including both the electron density ρ_*e*_(**r**) and the nuclear charges *Z_I_* at positions **R**_*I*_) is readily computed using the Coulomb law,
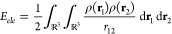
6where, for the sake of simplicity, the electrostatic potential in this and the rest of the equations is expressed in atomic units. Interestingly, the QTAIM real space partition derived from ρ_*e*_(**r**) allows us to decompose the electrostatic energy at the atomic level,
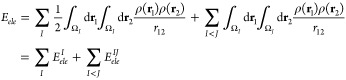
7

Similarly, the fragment-based decomposition can be readily accomplished in an analogous way, allowing thus the specific assessment of the electrostatic component of the formation energy Δ*E*_*ele*_ of a two-fragment system *AB* as
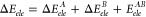
8where Δ*E*_*ele*_ is expressed in terms of two contributions, namely, the intrafragment variations of electrostatic energy in the formation process,  and , and the interfragment electrostatic interaction, . At this point, we note that although Δ*E*_*ele*_ is commonly termed as a classical electrostatic interaction energy, we will refer to it as the electrostatic contribution to the formation energy of the A···B complex in order to help avoid confusions with the IQA/IQF interaction energy terms. When the charge density is constructed from the unrelaxed fragment densities as , the electrostatic contribution to the formation energy, which is named here as the zeroth-order energy , equals the Coulomb interaction between the unrelaxed densities:
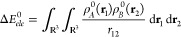
9This energetic term corresponds to the so-called *first-order polarization energy* (or simply *electrostatic energy*) defined in SAPT,^16^ which has been adopted as a benchmark electrostatic energy for the validation of recently developed short-range electrostatic potentials.

### 2.3 Electrostatic Potentials in Molecular Mechanics

To avoid the usage of continuous charge distributions, the MM methods typically invoke the multipolar expansion as detailed in the Supporting Information (SI), which approximates the zeroth-order energy defined in eq 9. Formally, the multipolar electrostatic energy  is affected by two different error sources. On the one hand, the underlying expansion must be truncated at some order (*l*_*max*_ = 0, 1, 2, ...), resulting thus in a certain truncation error. On the other, when  and  overlap to a significant extent, the rigorous application of the multipole expansion is impeded so that its usage at short distances implies some charge penetration error, which is normally assumed to be dominant. Nevertheless, the multipole-based potentials are still largely useful in many cases, and they enhance convergence by distributing multipoles throughout the molecule at the atomic sites and/or bond centers.^9,35,36^

The MM electrostatic potentials can be classified into two groups. On one side, MM methods such as AMBER,^37^ CHARMM,^38^ GROMOS,^39^ and OPLS^40^ adopt simple electrostatic formulas with point charges (i.e., monopoles, with *l*_*max*_ = 0) that are ultimately the result of a fitting procedure against the molecular electrostatic potential (ESP). On the other side, more sophisticated methods, such as NEMO,^41^ AMOEBA,^42^ or the QTAIM-based FFLUX,^43,44^ include higher order multipoles (frequently up to the quadrupoles, *l*_*max*_ = 2) in order to capture the anisotropy of the distribution of electrons in space. These potentials are generally built from the QM density matrix of the molecule of interest by means of the distributed multipole analysis (DMA)^36^ or similar procedures. In addition, some methods (e.g., AMOEBA or NEMO) also refine the DMA multipoles to better reproduce the ESP values. In this way, the resulting charges/multipoles may include in an effective way both high-order multipolar contributions and CP effects. Actually, the performance of the MM potentials is examined statistically as a whole (i.e., using the full MM potential including all bonded and nonbonded terms) by various energetic and structural validation tests. A quite different approach is followed by the FFLUX force field. It makes use of QTAIM multipoles in contrast to the more widespread DMA methodologies and estimates them by means of a machine learning technique depending on each atom’s environment.

In comparison with the atomic/multipolar methods that are massively employed in current simulation packages, the electrostatic MM potentials that go beyond the multipolar approximation are much less consolidated. In this category, we find different methodologies such as SIBFA,^45^ EFP,^46^ and AMOEBA+^24^ that complement the multipolar formulas with other (so-called damping) functions to capture very-short-range electrostatics and to remove the CP error. In this way, these potentials (whose general form is shown in the SI) seek to reproduce  as evaluated by SAPT or similar methodologies.^24^

### 2.4 Charge Penetration Energy

The CP energy *E*_*pen*_ between two molecules has been defined^47^ as the difference between the exact zeroth-order electrostatic energy  and its multipolar analogue ,
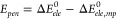
10

Conceptually, this straightforward definition of *E*_*pen*_ is satisfactory. It also shows that *E*_*pen*_ is not only an interfragment quantity but rather an energy that presents also intramolecular contributions according to the real space partitioning of the whole . In this respect, the energetic definition suggests that the CP energy is not limited to *the change in the electrostatic interaction between two atoms due to their electron cloud overlap and the associated loss of nuclear screening*.^48^

The rigorous evaluation of *E*_*pen*_ for different systems at varying intermolecular separations would allow a deeper analysis of electrostatics and, eventually, the development of more accurate potentials. However, as noticed by Bojarowski et al.,^47^*different methods of obtaining multipole moments lead to different radii of (pseudo)convergence, different levels of multipole expansions at which (pseudo)convergence is achieved, and different values of penetration energy*. Therefore, the value of the CP energy as evaluated with eq 10 may depend on the particular method used to derive the multipoles. Moreover, the usage of truncated expansions introduces some additional truncation error so that both truncation and penetration effects become somewhat mixed in the resulting *E*_*pen*_ values.^49^

An alternative to evaluate *E*_*pen*_ has been proposed by Kairys and Jensen.^50^ Having noticed the relationship between the CP energy and the magnitude of the orbital overlap, they attempt to recover such an effect from scratch, with a derivation of *E*_*pen*_ independently from the multipolar model used to estimate electrostatics at first stage. However, the authors find that the inherent dependence on the set of molecular orbitals used may lead to different CP values.

#### 2.4.1 A Novel IQF Definition of the Charge Penetration Energy

By combining both the Bader partitioning scheme ) with a total zeroth-order density decomposition (), the following energy terms are obtained:

(i) the intramolecular interaction due to  or  inside a given molecular basin Ω_*A*_ or Ω_*B*_, leading to , , , and .
(ii) the intramolecular interaction between the two monomeric densities inside a given basin:  and .
(iii) the intermolecular electrostatic energy between the same density pieces:  and .
(iv) the intermolecular interaction between  and  in opposite molecular basins:  and .

Hence, the total electrostatic energy of a complex *AB* can be written as
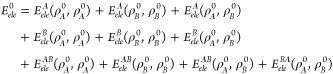
11In the notation used above the two interacting densities are encompassed by parentheses, while the basins they are integrated in are identified by the corresponding superscripts in the given order (only one if both are the same). Hence, for instance, the term  stands for  and  corresponds to .

When the above double decomposition is applied to the electrostatic energies of the separate fragments, such as *A*, in the final complex, the electrostatic energy of the original species becomes
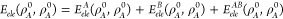
12

Note that this partitioning is derived from the *AB* zeroth-order (i.e., Hartree product) wave function and that geometry relaxation effects are not considered. By subtracting from eq 11 the previous fragment energies, the corresponding electrostatic contribution to the formation energy of the complex is obtained,
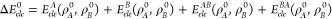
13

Among the surviving terms in eq 13,  reveals itself as the ordinary interaction term between the two monomers *A* and *B*. It matches  at long distances, while the other three terms would present a similar behavior of increasing in magnitude when shortening the intermolecular distances *R*_*AB*_ and canceling out in the opposite situation. Thus, those three terms can be directly related with the interpenetration of molecular densities and grouped in the IQF-like electrostatic charge penetration energy
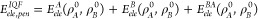
14

**Figure 1:**
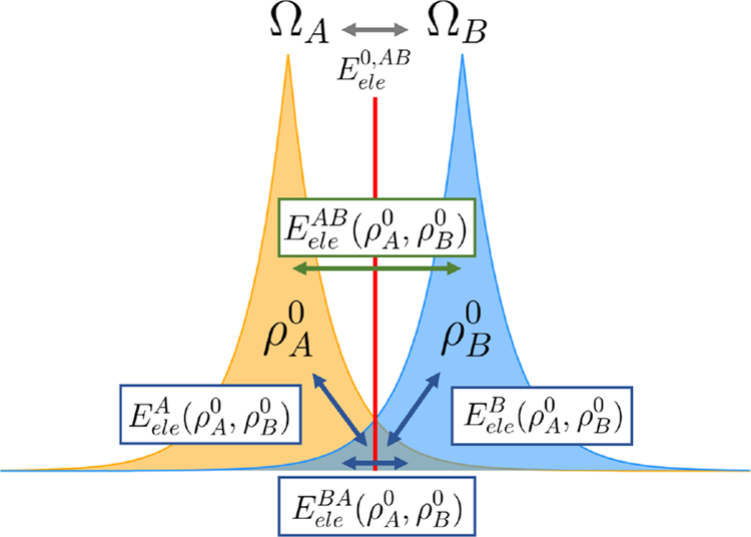
Graphical scheme of the four contributions giving rise to , where three of them (in dark blue) comprise the IQF electrostatic penetration energy and the remaining one (dark green) accounts for the interaction of  and  lying in the molecular basins Ω_*A*_ and Ω_*B*_, respectively. The zeroth-order IQF pairwise term  has been also included to highlight its difference with the previous , as it accounts for an interaction between total densities inside each basin (the original  or  and the tail from the other that has penetrated into another domain).

This term fulfills  (and so its three components), while . Figure 1 represents the previous four terms between the partitioned  and  adding up to  and compares them to the  term between the total densities in each basin.

## 3 Computational Details

### 3.1 Molecular Geometries and Reference Interaction Energies

All the QM and classical electrostatic calculations were performed on the molecular geometries retrieved from the S66 database,^29^ which contains a set of 66 complexes featuring the most common noncovalent interactions in biomolecules. These can be classified depending on the atoms involved into polar, nonpolar, and mixed. Analogously, the different complexes have been grouped into H-bond, dispersion, and mixed according to the main interactions they experience (see Table S1). For representing both the atomic interactions and the subsets of complexes, a color code has been utilized: magenta for H-bond/polar, yellow for mixed, and blue for dispersion/nonpolar. In addition to the S66 set, a selection of 12 representative complexes from the S66x8 database,^30^ which is an extension of the former to eight different fractions of the equilibrium intermolecular distances, were also considered. The benchmark CCSD(T)/CBS interaction energies collected in S66 were employed as the reference values for comparative purposes.

### 3.2 HF-D3 Calculations

HF/cc-pVTZ calculations were carried out on the S66 and the S66x8 geometries using the GAMESS-US package.^51^ Grimme’s D3 dispersion potential as implemented in the DFT-D3 code^52^ was employed to incorporate the dispersion energy. Additionally, in order to correctly reproduce the asymptotic behavior of the dispersion energy at small distances, the Becke–Johnson damping function was chosen.^53^

We selected HF because it lacks entirely dispersion energy and thereby yields a straight physical partitioning of energy in combination with the D3 potential. We also note in passing that HF-D3 has been shown to describe correctly and efficiently the structure and energetics of biomolecules^54^ and that a variant of DFT-SAPT has been also developed in which the costly *ab initio* dispersion calculations are replaced by a reparametrized D3 potential.^55^ In addition, the HF-D3/cc-pVTZ energies reproduce quite well the reference CCSD(T)/CBS energies of the S66 structures (see Figure S1).

### 3.3 IQA Energy Decomposition Analysis

The decomposition of the QM and the electrostatic energies derived from continuous charge densities were performed with the PROMOLDEN code.^56^ The integration settings comprised β-spheres with radii of 60% of the distance between each nucleus and its closest critical point. Within them, Lebedev angular grids with 974 points were used, along with Euler–McLaurin radial quadratures with 382 radial points. A bipolar expansion of  was selected with an *l*_*max*_ of 6. On the other hand, the outer part of the basins (i.e., outside the β-spheres) employed the same angular and radial quadratures, albeit increasing their respective points up to 5810 and 512, with a maximum radius of 15 au. In this case,  was expanded by means of a Laplace expansion with *l*_*max*_ = 10.

### 3.4 Point-Charge and Multipolar Calculations

Atomic charges were computed for the separate monomers in the S66 structures by means of the restrained electrostatic potential (RESP) method following the General Amber Force Field (GAFF)^57^ prescriptions with a HF/6-31G* level of theory. In the case of the atomic multipoles, two different sets were employed. On the one hand, AMOEBA multipoles were derived up to the quadrupoles (*l*_*max*_ = 2) following its corresponding parametrization protocol.^42,58^ On the other, QTAIM multipoles were obtained by means of the PROMOLDEN program with an *l*_*max*_ = 2. Both the AMOEBA and the QTAIM multipolar energies were obtained with the MPOLINT code.^59^

Additionally, a set of 12 S66x8 complexes was tested under the AMOEBA+ CP-corrected potentials.^24^ For this, TINKER was used to calculate the respective CP energies as the difference between the CP-corrected multipoles and the multipolar energies previously derived. The parameters of the damping functions were directly taken from the literature.^24^

### 3.5 Graphs and Statistical Analyses

Octave^60^ and GNUplot^61^ were, in turn, used to perform the statistical analyses and the correlation plots, while Python’s Matplotlib^62^ was chosen for the rest of the representations.

## 4 Results and Discussion

### 4.1 IQF-D3 Partitioning and Pairwise Approximation

**Figure 2:**
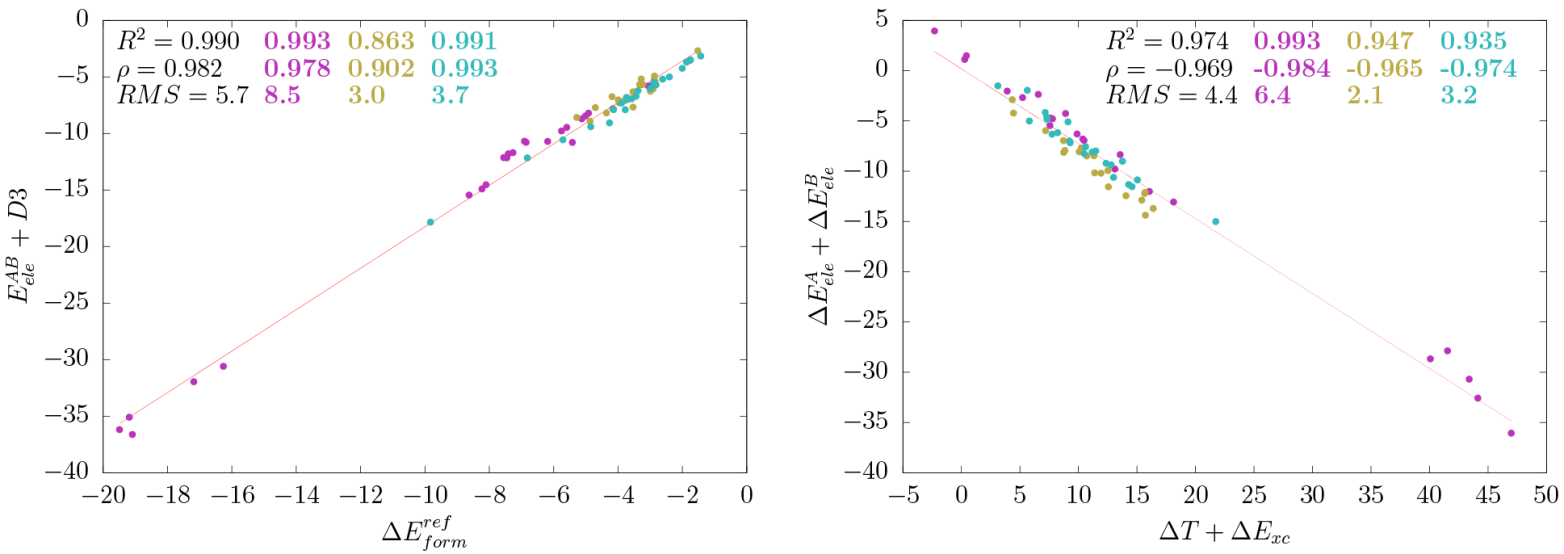
Left: correlation between the dispersion-augmented IQF intermolecular electrostatic energy  and the reference binding energies . Right: anticorrelation featured by the intrafragment electrostatic contribution to formation  and the total kinetic plus exchange-correlation contributions Δ*T* + Δ*E*_*xc*_. The statistical analysis comprises the coefficient of determination *R*^2^, Spearman’s rank correlation coefficient ρ, and the root-mean-square error *RMS*. Data corresponding to the whole set of complexes is depicted in black and that ascribed to the H-bond group is in magenta, while mixed and dispersion complexes are in yellow and blue, respectively. All the energies are in kcal mol^–1^.

The IQF-D3 decomposition of the HF/cc-pVTZ binding energies for the S66 complexes has been discussed at length in previous work.^32^ Herein, we focus on the decomposition of the electrostatic descriptors into intra- and interfragment components. Interestingly, we found that the combination of the interfragment electrostatic interaction energy  with the D3 dispersion potential yields pairwise energies that are quite well correlated with the S66 benchmark values, the coefficient of determination being *R*^2^ = 0.990 with *RMS* errors of 5.7 kcal mol^–1^ (see Figure 2 and Table S2). Thus, the IQF  descriptors in conjunction with the D3 potential capture the essential electrostatic and dispersion interactions that determine the relative stability of the noncovalent complexes. When addressing both terms independently (Figure S2), we find that the pure electrostatic  term exhibits a satisfactory overall correlation (*R*^2^ = 0.943) due to the fundamental role of electrostatics in H-bond complexes. On the other hand, the D3 descriptor has a null global correlation with the S66 reference energies, although it is reasonable (*R*^2^ = 0.820) for the dispersion complexes as expected. However, the mixed complexes are not well-described by either the electrostatic or the dispersion energies separately, and their combination becomes critical.

In contrast to the ability of the  descriptors to capture the main features of noncovalent binding, the combination of Δ*E*_*ele*_, which includes both the intra- and the intermolecular electrostatic effects, with the D3 potential deteriorates the global correlation (*R*^2^ = 0.888) and results in larger *RMS* errors (17.3 kcal mol^–1^). The full IQF decomposition (eq 8) explains this unbalanced description because the intrafragment electrostatic energies, which contribute to the deformation energies, tend to cancel out with the QM energy terms (electronic kinetic energy and exchange-correlation) that are not required in the simple electrostatic + dispersion picture (see Figure 2 right). Therefore, the pairwise  terms arise as the most relevant IQF electrostatic descriptors of noncovalent binding.

### 4.2 Validating and Analyzing the Zeroth-Order Approximation

The electrostatic IQF terms can be readily evaluated under the zeroth-order approximation (i.e., ). Thus, it turns out that the interaction energies  can be replaced effectively by their zeroth-order counterparts. Indeed, the pairwise  energies have low *RMS* errors (3.1 kcal mol^–1^) and maintain a good correlation (*R*^2^ = 0.971) with respect to the benchmark data (Table S3). This behavior is also satisfactory within the S66 subsets: *R*^2^ = 0.989 and 0.988 for the polar H-bonded systems and the dispersion-dominated complexes, respectively, albeit the correlation is somewhat reduced in the case of the mixed complexes (*R*^2^ = 0.755). Further support for the use of the zeroth-order energies comes from the atomic level, where a high degree of coincidence between the diatomic zeroth-order  and fully relaxed  energies is also found at the equilibrium geometries (*R*^2^ = 0.995, see the SI).

**Figure 3:**
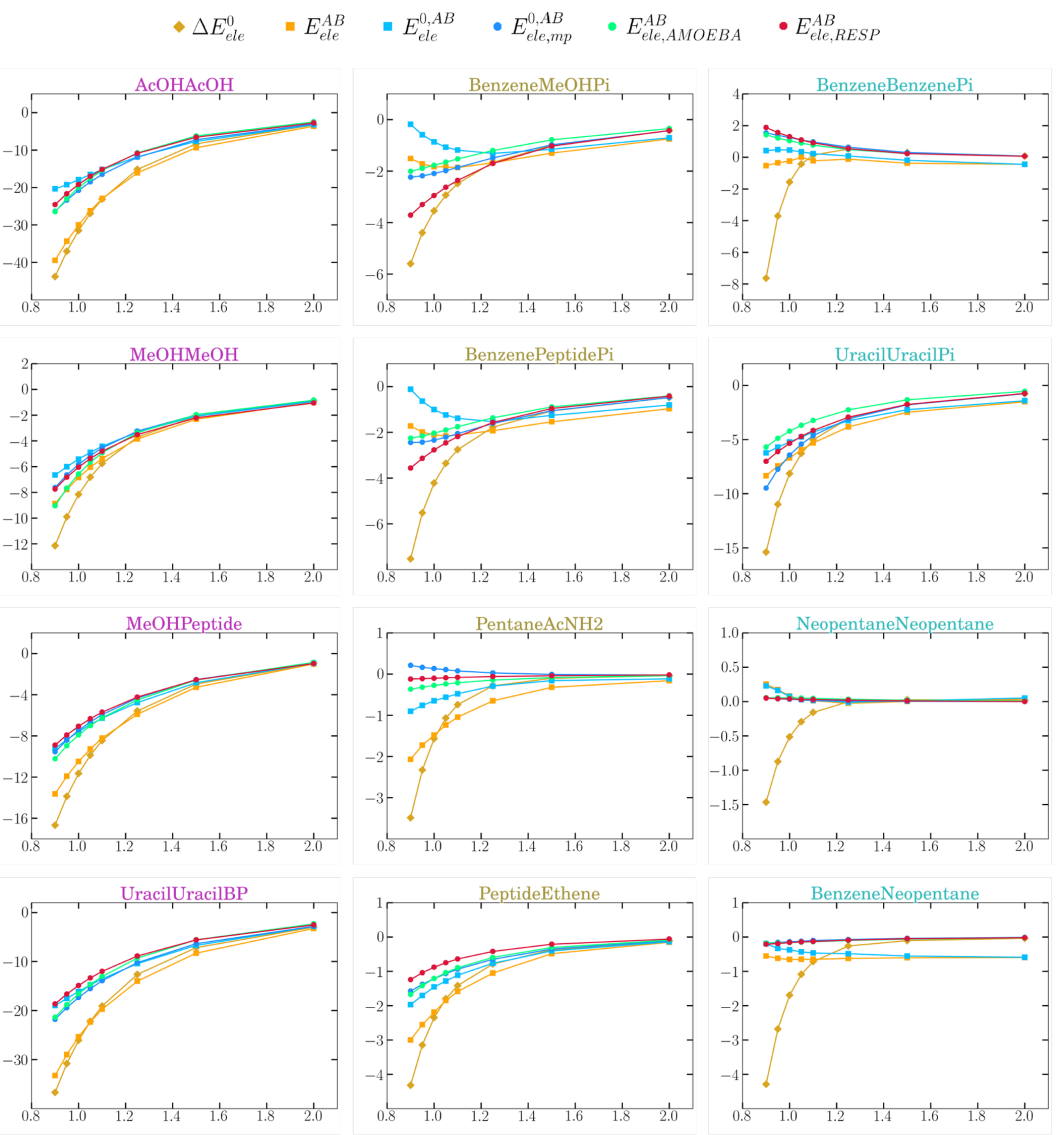
Intermolecular electrostatic interactions for a subset of the S66x8 complexes as provided by IQF (either exactly  or under the zeroth-order approximation ), zeroth-order QTAIM multipoles , AMOEBA multipolar energies , and RESP atomic charges . Additionally, the zeroth-order electrostatic contribution to formation  is also included. The complexes are colored and displayed in columns according to the group they belong to, namely, H-bond, mixed, or dispersion, respectively. The energies (*Y*-axis) are given in kcal mol^–1^, and the abscissas represent the intermolecular distances relative to the equilibrium ones (*R*_*AB*_/*R*_*eq*_).

When addressing the distance dependence of the previous term (see Figure 3), both  and  follow the same trends at varying intermolecular separations *R*_*AB*_ (given as relative to the equilibrium distances *R*_*eq*_). As expected, they start diverging at short distances due to the strengthening of charge polarization, charge-penetration, and charge-transfer effects that attenuate the pairwise electrostatic forces. The magnitude of these effects is clearly system-dependent, as well as the shape and slope of the  and  curves, revealing thus further details about the role of electrostatics in these complexes. Thus, the electrostatic stabilization of the four H-bond complexes and others (e.g., the π-complex of the uracil dimer) is continuously reinforced upon shortening the monomer–monomer distance, reflecting the major electrostatic control of these systems. In contrast, the T-shaped benzene complexes with methanol or *N*-methylacetamide reach an electrostatic minimum at a distance longer than the equilibrium one while the small electrostatic energies of the dispersion dimers (i.e., +1, −1 kcal/mol) change very little along the curves (some small leaps are due to residual errors arising in the numerical integration over the atomic basins).

In Figure 3 the deviation between the global  energies and the interfragment  anticipates the underlying CP effects associated with the density overlap. For the H-bond and some of the mixed complexes, the two curves decrease with lowering separation, but they split gradually for *R*_*AB*_/*R*_*eq*_ < 1.6. The global  stabilization nearly doubles  at *R*_*eq*_, showing thus the large impact of intramolecular electrostatics as defined in the IQF framework. For the π-complexes (benzene–dimer, benzene–methanol, ...) or the weakly interacting neopentane dimer, the inter- vs intramolecular balance is differently modulated because the deviation between the global and the interfragment electrostatics becomes significant only at very close distances (e.g., *R*_*AB*_/*R*_*eq*_ < 1.1), which are indicative of mutual overlap. In these systems,  is thus reinforced by several kcal mol^–1^, which are ascribed to the intrafragment electrostatic stabilization achieved by the fragment-overlap (i.e., CP) effects. Such effects have a minor influence on the small  energies (<1–2 kcal mol^–1^), which tend to remain nearly constant or become slightly attenuated. As shown below (Section 4.5), the IQF analysis of the CP energy gives further insight about the behavior of  and  with *R*_*AB*_/*R*_*eq*_.

### Comparison between  and Pairwise MM Energies

4.3

The pairwise approximation that emerges from the IQF-D3 decomposition and the validity of the zeroth-order approximation for the electrostatic interactions provide an insightful theoretical support for the construction of noncovalent MM potentials. In this scenario,  can be seen as the most suitable IQF descriptor to assess the approximate electrostatic potentials. Hence, we calculated the interfragment electrostatic energies using the RESP atomic charges and the AMOEBA multipoles, as well as the QTAIM multipoles up to the quadrupoles.

According to the statistical data in Table 1, either the RESP atomic charges or the QTAIM/AMOEBA multipoles give interfragment electrostatic energies that correlate considerably well with  (*R*^2^ > 0.9 and *RMS* errors ∼ 1 kcal mol^–1^) for the full S66 set and also for the H-bond/dispersion subsets. These point-charge/multipolar electrostatic energies are less satisfactory for the less abundant mixed complexes, although the multipolar potentials yield a more accurate description (*R*^2^ ≃ 0.6–0.8) than the RESP charges (*R*^2^ ≃ 0.5). In addition to  and the fully relaxed and zeroth-order IQF pairwise terms, Figure 3 also displays the distance dependence of the QTAIM/AMOEBA/RESP energies, that results quite close to that of the interfragment  energies. Nevertheless, a closer inspection reveals that the QTAIM/AMOEBA/RESP energies tend to overestimate the stabilizing/destabilizing character of  for the H-bond/dispersion dimers, respectively.

**Table 1:** Statistical Measurements Comprising the Coefficient of Determination *R*^2^, Spearman’s Rank Correlation Coefficient ρ, and the Root Mean Square Error *RMS* for the Correlation between  and either the QTAIM or AMOEBA Multipoles (*l*_*max*_ = 2) or the RESP Point Charges (*l*_*max*_ = 0)

multipolar approximation	complex type	*R*^2^	ρ	*RMS*
QTAIM	global	0.970	0.958	1.0
	H-bond	0.956	0.904	1.4
	mixed	0.644	0.768	0.9
	dispersion	0.955	0.795	0.5
				
AOMEBA	global	0.953	0.972	1.3
	H-bond	0.904	0.841	2.0
	mixed	0.800	0.845	0.7
	dispersion	0.939	0.893	0.4
				
RESP	global	0.974	0.962	0.8
	H-bond	0.981	0.918	0.7
	mixed	0.456	0.687	1.1
	dispersion	0.948	0.831	0.3

The good agreement between the multipolar and the RESP energies in Table 1 and in Figure 3 suggests that the RESP fitting procedure may incorporate in an effective way higher order effects even at short distances. In addition, our results point out that the pure QTAIM multipoles can be employed in the construction of accurate electrostatic potentials, free from the inclusion of other effects that may be present when the DMA multipoles are fitted against the molecular ESP. In fact, the QTAIM multipoles, which are already considered in the FFLUX force field, readily reproduce the ESP without the need of any constraint.^63^

### 4.4 Comparing Diatomic Electrostatic Interactions

IQA permits an unambiguous decomposition of the continuous-density intermolecular interaction energy into a sum of atomic and diatomic terms that enables a thorough analysis of the global molecular properties based on their atomic origins, and a close comparison with the various MM descriptions at this atomic level.

**Figure 4:**
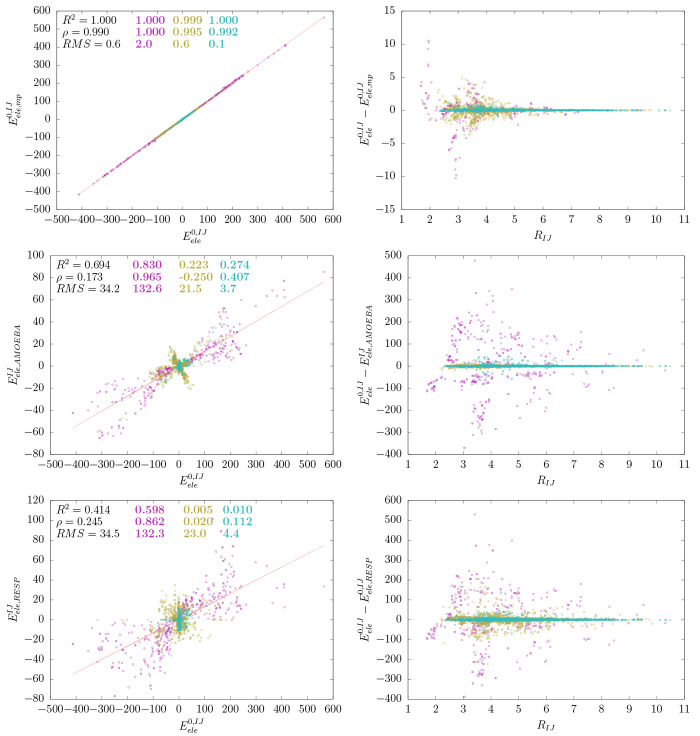
Comparison of the  and  energies with the  term (kcal mol^–1^). On the left are the correlation plots and, on the right, each difference as a function of the interatomic distance (Å).

As expected, the IQA diatomic terms correlate almost perfectly with the QTAIM multipolar ones  (see Figure 4). On the contrary, the AMOEBA and RESP energies are significantly less correlated (*R*^2^ of 0.7 and 0.4, respectively) and have large *RMS* errors. For example, the largest discrepancies between  and the QTAIM-multipolar  in the acetic acid dimer (about 6 kcal mol^–1^) arise from the atoms involved in the OH·O H-bonds, the rest of pair interactions having much lower differences (<0.5 kcal mol^–1^; see Tables S7–S9). When comparing  and  (or ), the largest discrepancies amount to hundreds of kcal mol^–1^ and involve not only short polar contacts but methyl C atoms too (see Tables S10–S15).

The dissimilarity between the  energies and the / values was not entirely unexpected given that the RESP charges are derived from the molecular ESP and the AMOEBA multipoles are obtained by the DMA protocol. In fact, a difference of 1 order of magnitude between the atom–atom electrostatic interactions from IQA and MM potentials has also been noticed previously.^64^ The present results show in further detail the actual discrepancies between the various atomic representations and suggest that, although the diverse atomic multipoles employed in classical potentials yield similar molecular electrostatic energies, the atomic decomposition is more questionable, which, in turn, can negatively affect the interpretation of localized electrostatic interactions and/or result in artifacts while dealing with QM and MM short-range electrostatics in hybrid QM/MM methodologies.

### 4.5 Charge Penetration under the QTAIM Scrutiny

Following the prescriptions introduced in Theory and Methods, the zeroth-order electrostatic formation energy  of each S66 complex was decomposed by combining its real space partition into nonoverlapping atomic basins with the zeroth-order density approximation (). This strategy leads to the IQF-based charge penetration energies, , resulting from the sum of the intramolecular terms  and , as those accounting for the interaction of both densities inside the same basin, and the intermolecular energy  between the tails of each molecular density that penetrate into the opposite basin, as described in eq 14. This constitutes an *effective* penetration energy in the sense that the molecular identity between two overlapping fragments becomes necessarily blurred so that fragment properties are dependent upon the scheme followed to dissect the global charge density into its constituents. Nevertheless, the topological analysis of ρ^0^ yields a consistent identification of molecular fragments so that we believe that the associated charge-penetration analysis can give useful insight into the electrostatics of noncovalent complexes.

**Figure 5:**
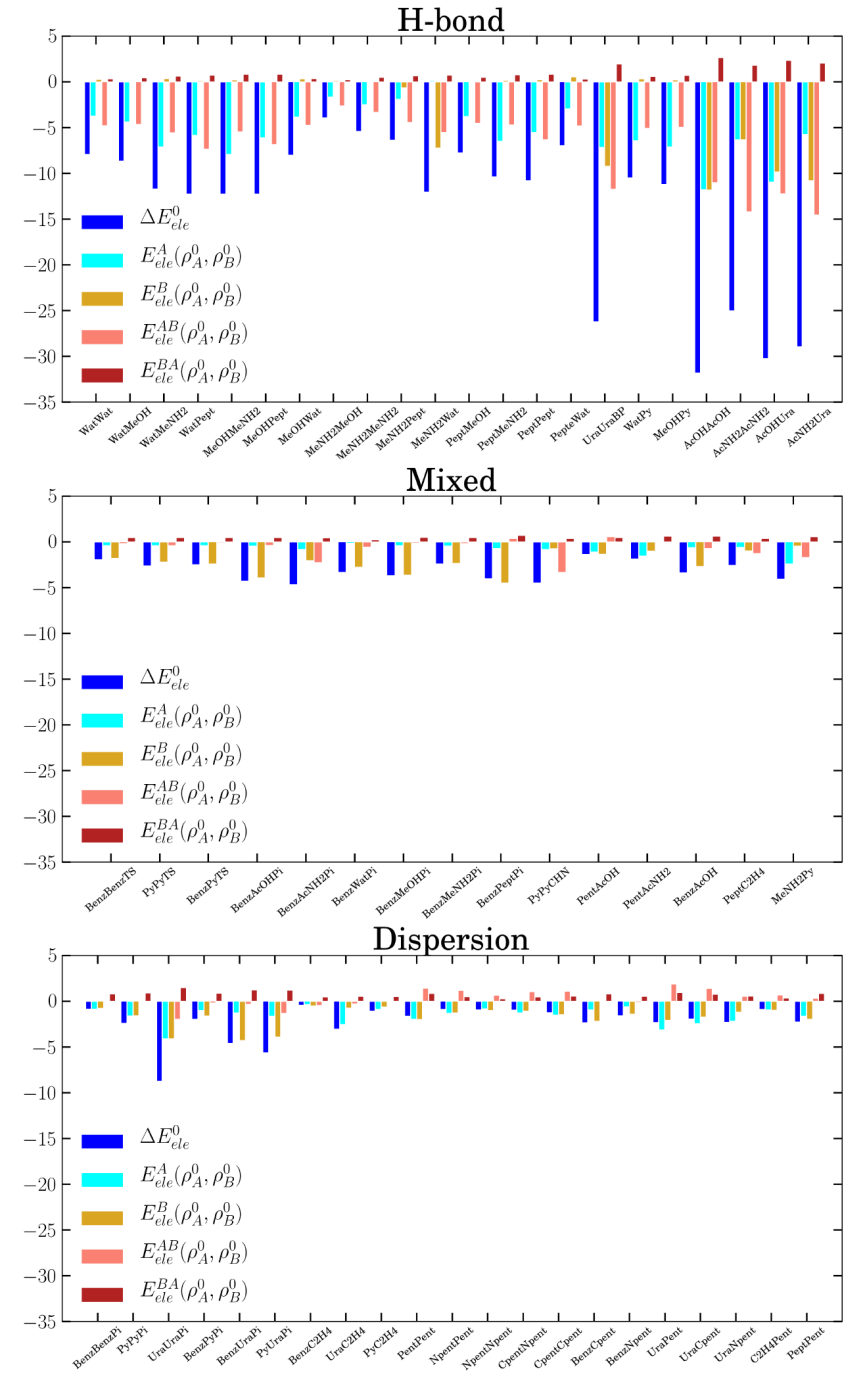
Decomposition of  into  and the three IQF penetration terms , , and . Energies are given in kcal mol^–1^.

The application of eq 14 to  results in the energy contributions shown in Figure 5. On the one hand, the interfragment energy  is formally not affected by charge penetration and plays a stabilizing role in all the H-bond complexes (slightly repulsive in the dispersion complexes). On the other hand, the IQF penetration term  turns out to be of equal importance in the H-bond complexes or even more relevant in the dispersion subset for which penetration energy describes the major part of .

The decomposition of the penetration energy shows that it arises mainly from the stabilizing interactions between  and  inside the same basin. This is an *intramolecular* effect as reflected by the magnitude of the  and  energies. As shown by the integration of  or  in the corresponding basins, the mutual CP values range, for instance, from 0.035 e in the neopentane dimer to 0.099 e in the case of the acetic acid dimer. These fractional charges involve the e–e repulsion between the fragment electron densities occupying the same space, such as  such that **r** ∈ Ω_*A*_ (or equivalently in region Ω_*B*_), and the attraction experienced by the nuclei of one fragment  (or ) and the fraction of electrons from the other that has penetrated into the former  (or similarly ). In light of these results, e–n attraction greatly overcomes e–e repulsion between different zeroth-order densities inside the same basin and gives rise to the significant stabilizing energies observed. There is also a minor repulsive contribution owing to the purely electronic repulsion between the penetrating  into Ω_*B*_ and the  tail in Ω_*A*_, which is measured by .

**Figure 6:**
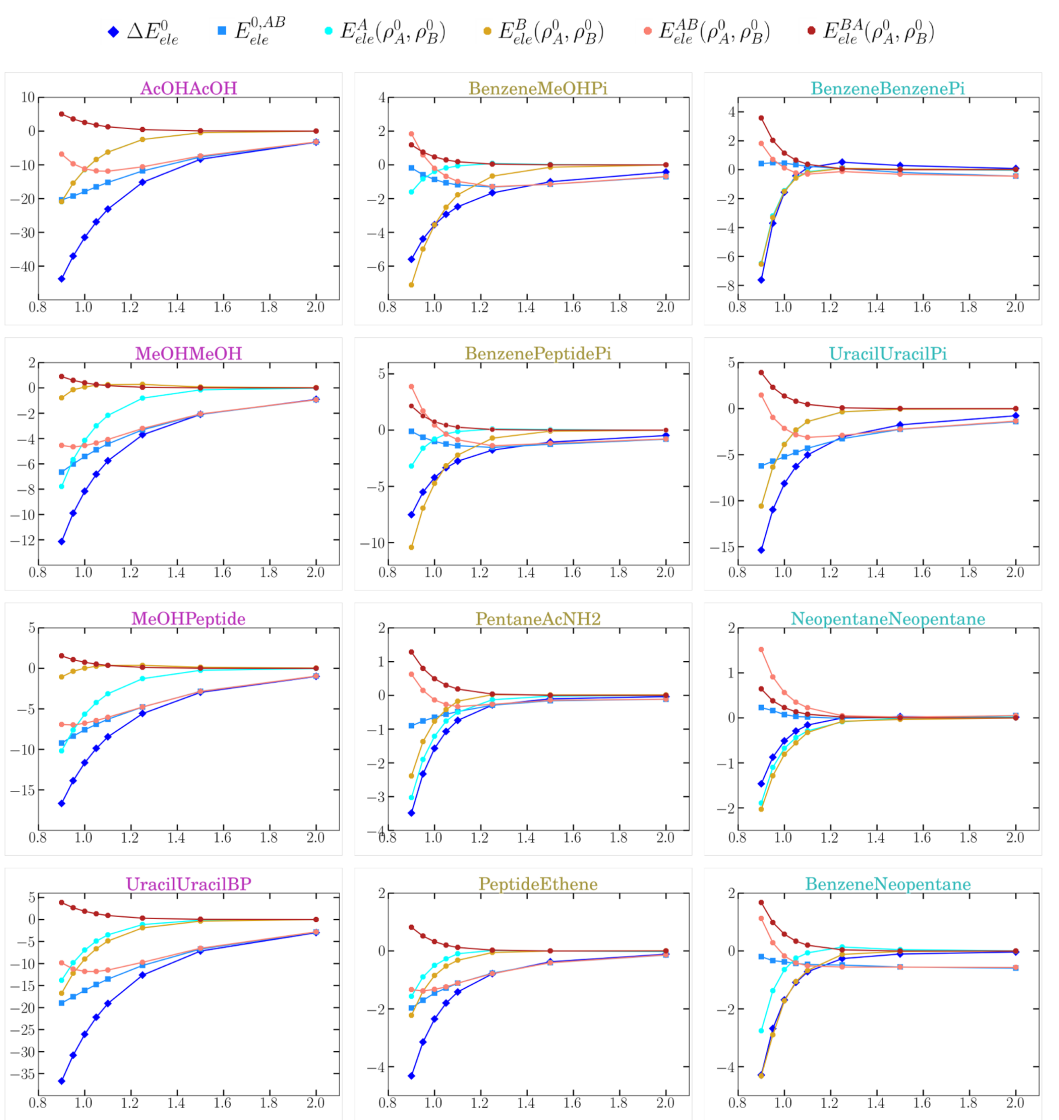
Evolution of the energy terms from eq 13, along with the  pair term as a function of the distance for the set of S66x8 systems chosen. The complexes are grouped in three columns as belonging to the H-bond, mixed, or dispersion subsets, respectively.

Further insight can be gained by analyzing the distance dependence of the various energy terms as shown in Figure 6. The plots confirm that the three components of  tend to zero when *R*_*AB*_/*R*_*eq*_ > 1.5 and further highlight the role of the intrafragment terms. Interestingly, the  energy, formally lacking penetration effects, is modulated by the degree of the interfragment overlap so that the decreasing trend in  is damped out or inverted at the shortest distances. This is not entirely unexpected given that, as two initially separated atomic basins (e.g, Ω_*I*∈*A*_ and Ω_*J*∈*B*_) approach one another, their volume, shape, and electron population evolve along the *R*_*AB*_/*R*_*eq*_ curve in response to the density overlap. We note, however, that the deviation of  with respect to the interfragment electrostatic energy  may constitute a useful index about the specific impact of penetration effects on the pairwise electrostatics. At this point, an important caveat should be noted. Within the QTAIM framework, the  energy includes a fraction of stabilizing penetration energy for *R*_*AB*_/*R*_*eq*_ < 1.2 given that the loss of some electronic  density from the basins of the monomer *A* is partially compensated by the penetration of  into the same basin. The fixed multipoles/charges in the classical potentials somehow mimic this behavior so that they remain closer to the  descriptors than to  around the equilibrium distance.

**Figure 7:**
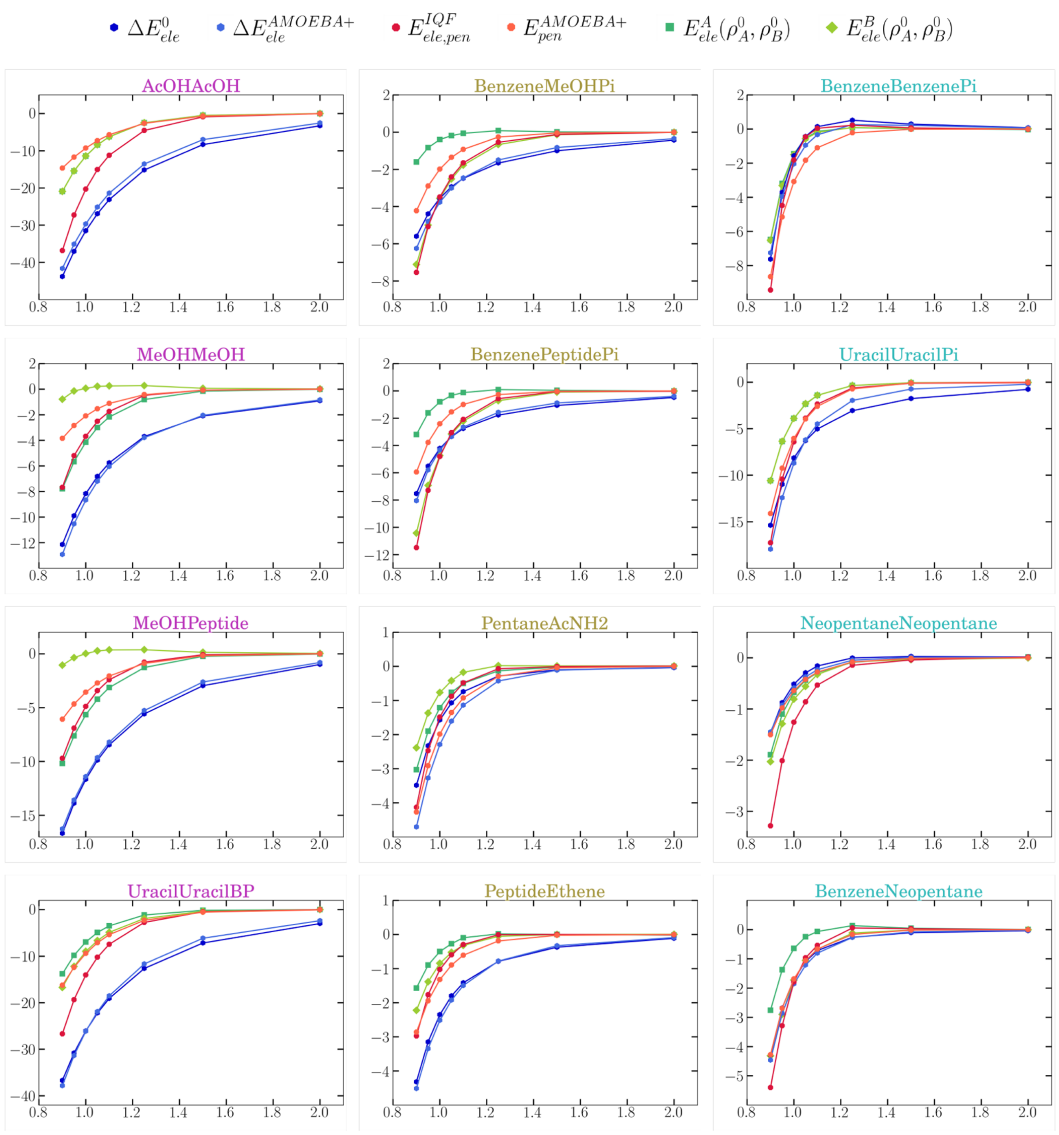
Comparison between the AMOEBA+ model and the zeroth-order IQF energies for our model S66x8 complexes. The complexes have been displayed according to the group they belong to (either H-bond, mixed, or dispersion). Distances (*X*-axis) are relative to the equilibrium ones (*R*_*AB*_/*R*_*eq*_) and energies (*Y*-axis) are in kcal mol^–1^.

Finally, Figure 7 compares the IQF penetration term and other relevant energetic quantities with the analogue term derived from the AMOEBA+ model as a function of the intermolecular distance. Thus, the combination of the multipolar  energies with the CP correction^24^ results in the  energies that approach to the reference , which is equivalent to the SAPT electrostatic energy. In effect, Figure 7 shows that  nearly matches . Concerning the CP energies, it is important to note again that the AMOEBA+ reference for measuring the CP energy is different from that provided by the IQF-QTAIM approach. Nevertheless, the two penetration energies exhibit a similar behavior with *R*_*AB*_, particularly for the more stable H-bond complexes, which resemble also the variations experienced by the intramolecular CP terms,  and . Therefore, we conclude that the AMOEBA+ CP and similar corrections account mainly for intramolecular electrostatics.

## 5 Concluding Remarks

In this work we have analyzed the short-range electrostatic interactions in the S66 and S66x8 data sets through a hierarchy of approximations at both the molecular and the atomic levels. We have shown first that the IQA/IQF decomposition augmented with the D3 dispersion terms gives support to the pairwise approach adopted by many MM potentials. In this respect, the interfragment energies  derived from the IQF partitioning suffice to capture the essential electrostatic effects explaining the binding of the weakly interacting complexes. Moreover, the same role can be played by the equivalent  values, which are obtained from the unrelaxed densities of the isolated monomers (i.e., the zeroth-order approximation).

According to our results, the intermolecular  energy turns out to be the most appropriate IQF descriptor to analyze and/or compare with electrostatic MM potentials. In particular, we have considered two widely used potentials relying on the RESP atomic charges or the AMOEBA distributed atomic multipoles, respectively, as well as the multipolar potential up to the quadrupoles derived directly from the QTAIM basins. The three MM pairwise approximations correlate satisfactorily with the zeroth-order IQF term at varying intermolecular distances and exhibit small *RMS* errors. However, when the  values are further decomposed into diatomic contributions, large discrepancies between the RESP or the AMOEBA atom–atom interactions and their zeroth-order IQA counterparts are unveiled. Although this is understandable in terms of the specific details of the RESP/AMOEBA charge/multipole derivations, it contrasts sharply with the nearly perfect match between the QTAIM atomic multipolar energies and the IQA reference values. Hence, MM potentials based on the QTAIM multipoles—such as the QCT-based FFLUX—may provide a more consistent description of electrostatic interactions at both the molecular and the atomic levels.

Besides forging links between the IQF/IQA quantities and the MM electrostatic potentials, we have studied the charge penetration effects that arise from the mutual interpenetration of the zeroth-order molecular densities in their opposite QTAIM basins as built from the final ρ^0^ of the complex. This QTAIM perspective allows us to dissect the CP energy into different contributions that emphasize its intramolecular character, which, in turn, is dominated by the attraction between the nuclei of fragment *A* (*B*) and the penetrating tail of density *B* (*A*). In this way we may clarify some practical issues related with the CP corrections for MM potentials. For example, adding CP corrections to MM potentials like RESP/AMOEBA, which target the zeroth-order interfragment electrostatic energy, results, necessarily, in an unbalanced description. This aspect, which has been overlooked in previous works,^10,48,65^ implies also that the electrostatic energy employed in popular MM force fields (AMBER, CHARMM, ...) cannot be compared with the global  energy derived from continuous charge distributions, but with its interfragment component. On the other hand, CP corrections have been derived to improve the description of the QM–MM electrostatic interactions in hybrid QM/MM methodologies.^12^ In this case, such corrections should mitigate short-range electrostatic artifacts, particularly those associated with the QM–MM covalent linkages. However, considering the highly dissimilar interatomic electrostatic energies produced by the QM densities and the RESP/AMOEBA potentials, the usage of electrostatic parameters more akin to the QM densities at the atomic level may have a larger impact in improving the QM–MM electrostatics.

Finally, concerning the novel MM potentials inspired by the QM SAPT methodology, it is clear that the multipolar electrostatics (interfragment) must be augmented by the CP potentials (intrafragment) if one seeks to reproduce the global electrostatics . Nevertheless, the IQF/IQA approach (and other EDAs) points out that the intramolecular electrostatic energy is closely related with other energy changes induced by fragment overlap (e.g., deformation and interfragment exchange-correlation energy), suggesting thus that the separate treatment of these effects by means of independent potential terms might be inefficient and hamper parameter development and transferability.

## Supporting Information Available

(1) Multipole expansion formulation, (2) a brief description of the CP corrections, (3) classification of the S66 complexes, (4) HF-D3–CCSD(T) comparison, (5) statistical analysis of the fully relaxed and zeroth-order electrostatic descriptions, (6) correlation study between the interatomic  and  terms, and (7) atomic interactions within the acetic acid dimer (PDF)
