# Supplementary material for: A Quantum Chemical Topology Picture of Intermolecular Electrostatic Interactions and Charge Penetration Energy
Source: J Chem Theory Comput. 2021 Jul 19;17(8):4981–95. doi: 10.1021/acs.jctc.1c00263 (PMC8901103; doi:10.1021/acs.jctc.1c00263)
Supplement: Supplementary file 1 — ct1c00263_si_001.pdf [file ct1c00263_si_001.pdf]

A Quantum Chemical Topology Picture of Intermolecular Electrostatic Interactions and Charge Penetration Energy: Supporting Information

Fernando Jiménez-Grávalos* and Dimas Suárez*

Dpto. de Química Física y Analítica, Universidad de Oviedo, E-33006, Oviedo, Spain.

E-mail: jimenezfernando@uniovi.es; dimas@uniovi.es

Contents

1 Multipole expansion **2**

2 Charge penetration corrections in MM potentials **3**

3 Classification of complexes **4**

4 HF-D3 vs. benchmark CCSD(T) energies **5**

5 Statistical analysis of electrostatic descriptors **5**

6 Correlation between diatomic E_{ele}^{IJ} and $E_{ele}^{0,IJ}$ energies **8**

7 Example of atomic-level comparison between $E_{ele}^{0,IJ}$ and its E_{ele}^{IJ}, $E_{ele,mp}^{0,IJ}$, $E_{ele,AMOEBA}^{IJ}$ and $E_{ele,RESP}^{IJ}$ analogues **9**

References **20**

Unless different units are specified, the data presented in the different sections of the Supporting Information is given in kcal mol^{-1} in the case of the energies and in Å for the distances.

1 Multipole expansion

Using spherical coordinates, the multipole expansion transforms Eq. 9 as

$$\Delta E^0_{ele,mp} = \sum_{l_1 m_1}^{\infty} \sum_{l_2 m_2}^{\infty} C_{l_1 m_1 l_2 m_2}(\hat{R}) \frac{Q^{0,A}_{l_1 m_1} Q^{0,B}_{l_1 m_1}}{R^{l_1+l_2+1}_{AB}}, \tag{S1}$$

where $Q^{0,A}_{l_1 m_1}$ and $Q^{0,B}_{l_2 m_2}$ are the multipoles of the respective unrelaxed densities $\rho^0_A(\mathbf{r}_1)$ and $\rho^0_B(\mathbf{r}_2)$.[1] In the previous Equation, m_i runs from $-l_i$ to $+l_i$, $R_{AB} = |\mathbf{R}_B - \mathbf{R}_A|$ is the separation between the centers of both charge distributions and $\hat{R}$ is the angular coordinate that determines the orientation. $C_{l_1 m_1 l_2 m_2}(\hat{R})$ are known coefficients calculated for each $(l_1 m_1, l_2 m_2)$ quartet. The set of multipoles Q^0_{lm} is obtained from the continuous charge density through the real spherical harmonics S_{lm} following the integral

$$Q^0_{lm} = N_l \int_{\mathbb{R}^3} r^l S_{lm}(\hat{r}) \rho^0(\mathbf{r}) d\mathbf{r}, \tag{S2}$$

N_l being a constant dependent on l, and r and $\hat{r}$ denote the radial and the angular coordinates, respectively.

The convergence of the multipolar expansion requires that the distances from the origin of each multipole series r_1 and r_2 cannot intercept each other, that is, $r_1 + r_2 < R_{AB}$. Within QTAIM,[2] atomic and, similarly, fragment multipoles are calculated from the corresponding atomic densities, which are non-overlapping by definition —i.e., they correspond to the extent of ρ inside each QTAIM basin. However, the $\Delta E^0_{ele,mp}$ energy computed from the QTAIM multipoles is also affected by the CP error. In fact, the well-defined atomic boundaries are often sharp and the convergence spheres defined by the outermost distances r_1 and r_2 from

each atomic position intercept each other. This explains the good agreement between the multipolar interatomic energies and those calculated by integration for relatively distant atoms, but not for bonded or very close pairs.[1,3,4]

2 Charge penetration corrections in MM potentials

In order to account for charge penetration, a damping function f_{damp} is usually applied to the multipolar electrostatic potential V_{mp} so as to estimate a CP-corrected one $\tilde{V}_{ele}$:

$$\tilde{V}_{ele} = V_{mp} \cdot f_{damp}(r). \tag{S3}$$

The functional form of the damping function is generally based on the correction to the hydrogen-like atomic potential that the expansion of r^{-1} does not include[5–8]

$$f_{damp}(r) = 1 - ce^{-\alpha r}, \tag{S4}$$

where c may be a constant or even a polynomial[9] dependent on the atomic separation r, and α is another parameter.

3 Classification of complexes

The complexes contained in the S66 dataset have been grouped in three sets according to the main interactions featured.

Table S1: S66 complexes classification. These are identified by their labelling in the data set.

H-bond	Mixed	Dispersion
4113_01WaterWater	4159_47BenzeneBenzeneTS	4136_24BenzeneBenzenepipi
4114_02WaterMeOH	4160_48PyridinePyridineTS	4137_25PyridinePyridinepipi
4115_03WaterMeNH2	4161_49BenzenePyridineTS	4138_26UracilUracilpipi
4116_04WaterPeptide	4162_50BenzeneEthyneCHpi	4139_27BenzenePyridinepipi
4117_05MeOHMeOH	4163_51EthyneEthyneTS	4140_28BenzeneUracilpipi
4118_06MeOHMeNH2	4164_52BenzeneAcOHOHpi	4141_29PyridineUracilpipi
4119_07MeOHPeptide	4165_53BenzeneAcNH2NHpi	4142_30BenzeneEthene
4120_08MeOHWater	4166_54BenzeneWaterOHpi	4143_31UracilEthene
4121_09MeNH2MeOH	4167_55BenzeneMeOHOHpi	4144_32UracilEthyne
4122_10MeNH2MeNH2	4168_56BenzeneMeNH2NHpi	4145_33PyridineEthene
4123_11MeNH2Peptide	4169_57BenzenePeptideNHpi	4146_34PentanePentane
4124_12MeNH2Water	4170_58PyridinePyridineCHN	4147_35NeopentanePentane
4125_13PeptideMeOH	4171_59EthyneWaterCHO	4148_36NeopentaneNeopentane
4126_14PeptideMeNH2	4172_60EthyneAcOHOHpi	4149_37CyclopentaneNeopentane
4127_15PeptidePeptide	4173_61PentaneAcOH	4150_38CyclopentaneCyclopentane
4128_16PeptideWater	4174_62PentaneAcNH2	4151_39BenzeneCyclopentane
4129_17UracilUracilBP	4175_63BenzeneAcOH	4152_40BenzeneNeopentane
4130_18WaterPyridine	4176_64PeptideEthene	4153_41UracilPentane
4131_19MeOHPyridine	4177_65PyridineEthyne	4154_42UracilCyclopentane
4132_20AcOHAcOH	4178_66MeNH2Pyridine	4155_43UracilNeopentane
4133_21AcNH2AcNH2		4156_44EthenePentane
4134_22AcOHUracil		4157_45EthynePentane
4135_23AcNH2Uracil		4158_46PeptidePentane

4 HF-D3 vs. benchmark CCSD(T) energies

Figure S1: Correlation between the HF-D3/cc-pVTZ (ΔE_{form}) and reference CCSD(T)/CBS (ΔE_{form}^{ref}) formation energies.

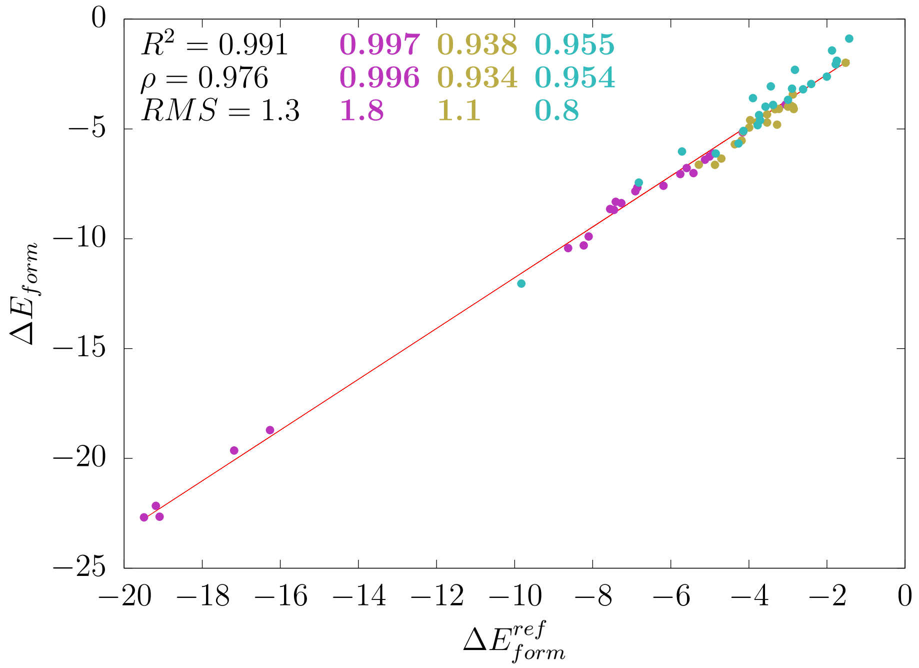

5 Statistical analysis of electrostatic descriptors

In order to attain a successful association of the IQF terms with binding we have inspected the different energy terms of Eq. 8 along with the D3 dispersion correction as compared with the S66 reference formation energies ΔE_{form}^{ref}. For this, the fully relaxed and the zeroth-order densities have been used. The data corresponding to each analysis are collected in Tables S2 and S3.

Table S2: Statistical analysis for the correlation between the benchmark ΔE^{ref}_{form} energies and the different fully-relaxed electrostatic contributions to formation augmented with D3 dispersion. The statistical descriptors comprise the coefficient of determination R^2, the Spearman correlation coefficient ρ and the root mean square errors RMS.

Energy term	Complex type	R^2	ρ	RMS
$\Delta E_{ele} + D3$	Global	0.888	0.719	17.3
	H-bond	0.970	0.930	24.0
	Mixed	0.593	0.820	12.8
	Dispersion	0.851	0.910	11.7
$E^{AB}_{ele} + D3$	Global	0.990	0.982	5.7
	H-bond	0.993	0.978	8.5
	Mixed	0.863	0.902	3.0
	Dispersion	0.991	0.993	3.7
ΔE^{A}_{ele}	Global	0.384	0.294	3.2
	H-bond	0.805	0.667	4.3
	Mixed	0.347	0.577	3.1
	Dispersion	0.375	0.563	1.7
ΔE^{B}_{ele}	Global	0.526	0.342	2.6
	H-bond	0.854	0.860	2.8
	Mixed	0.007	0.023	2.6
	Dispersion	0.029	0.388	2.4

Table S3: Comparison between the benchmark ΔE^{ref}_{form} energies and the zeroth-order electrostatic contributions to formation enhanced with D3 dispersion.

Energy term	Complex type	R^2	ρ	RMS
$\Delta E^{0}_{ele} + D3$	Global	0.956	0.898	7.1
	H-bond	0.994	0.983	9.4
	Mixed	0.121	0.684	5.1
	Dispersion	0.862	0.888	5.7
$E^{0,AB}_{ele} + D3$	Global	0.971	0.956	3.1
	H-bond	0.989	0.971	3.5
	Mixed	0.755	0.845	2.2
	Dispersion	0.988	0.992	3.3
$\Delta E^{0,A}_{ele}$	Global	0.325	0.656	4.9
	H-bond	0.599	0.782	7.0
	Mixed	0.160	0.204	3.5
	Dispersion	0.001	0.601	3.2
$\Delta E^{0,B}_{ele}$	Global	0.607	0.518	5.1
	H-bond	0.839	0.865	7.7
	Mixed	0.458	0.636	2.4
	Dispersion	0.163	0.759	3.1

The usage of the combined IQF-D3 strategy can be further illustrated by performing an individual analysis of the electrostatic and pure dispersion terms. As an example, the E_{ele}^{AB} and D3 dispersion correction are compared with the benchmark energies in Figure S2.

Figure S2: Correlation between ΔE_{form}^{ref} and either E_{ele}^{AB} (left) or D3 dispersion energy (right).

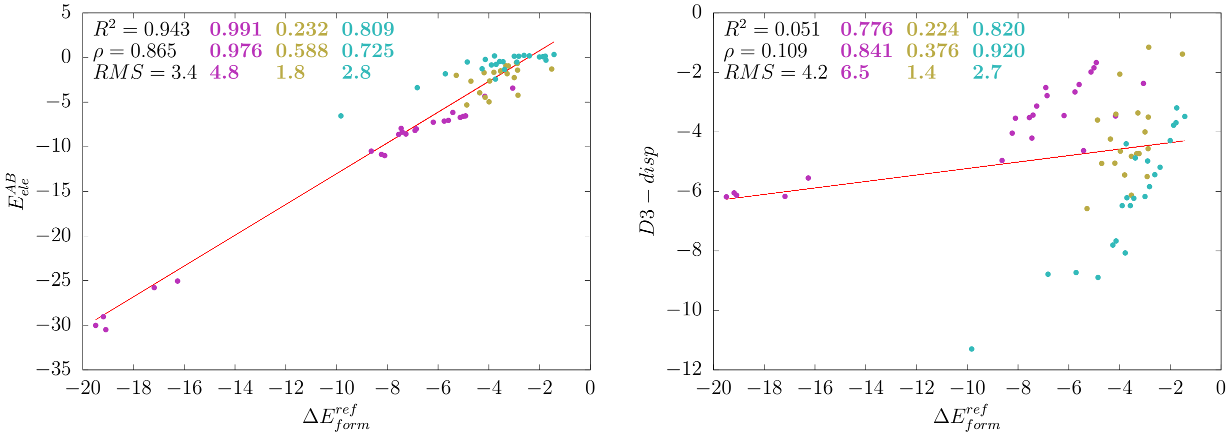

6 Correlation between diatomic E_{ele}^{IJ} and $E_{ele}^{0,IJ}$ energies

Figure S3: Left: Correlation between the exact atomic interactions E_{ele}^{IJ} and those subject to the zeroth-order approximation $E_{ele}^{0,IJ}$ with their corresponding statistical measurements. Right: Deviation of $E_{ele}^{0,IJ}$ with respect to E_{ele}^{IJ} as a function of the interatomic distance R_{IJ}.

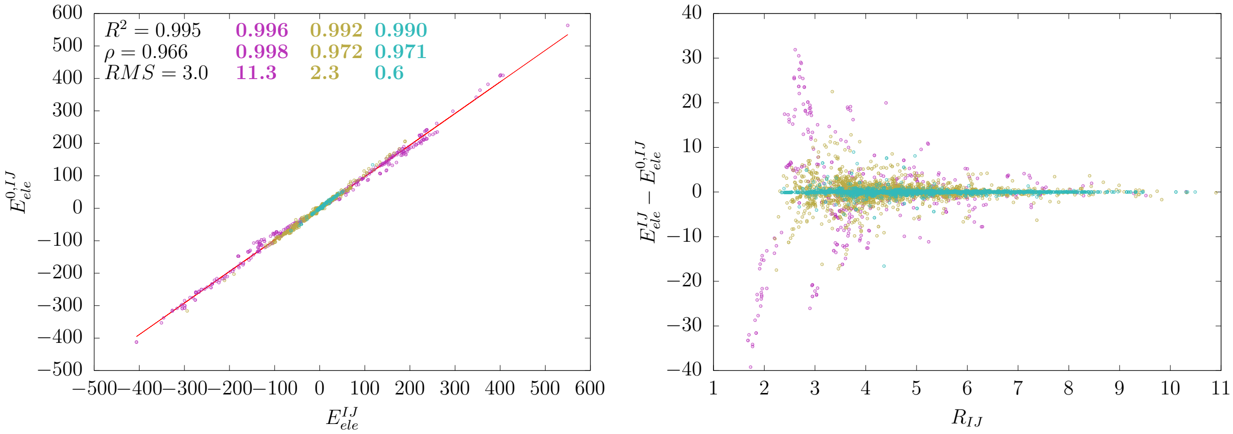

Figure S3 shows a high level of coincidence between the atomic zeroth-order $E_{ele}^{0,IJ}$ energy and its fully-relaxed counterpart E_{ele}^{IJ} ($R^2 = 0.995$). Only in the case of polar contacts the RMS error is relatively high (>10 kcal mol^{-1}), but the overall RMS remains about 3 kcal mol^{-1} due to the larger abundance of weak contacts. When considering the atomic sources of error, the plot on the right helps clarify the role played by the different kind of contacts in the global RMS value. Short polar contacts (e.g., O-H···O H-bonds) reveal as the major source of error. The atom-atom interactions are sometimes a few orders of magnitude higher than the interfragment energies, what highlights the well-known relevance of error cancellation in computing a global quantity. Tables S4-S6 present the atomic decomposition of both E_{ele}^{AB} and $E_{ele}^{0,AB}$ in the case of the acetic acid dimer to further illustrate this effect.

7 Example of atomic-level comparison between $E_{ele}^{0,IJ}$ and its E_{ele}^{IJ}, $E_{ele,mp}^{0,IJ}$, $E_{ele,AMOEBA}^{IJ}$ and $E_{ele,RESP}^{IJ}$ analogues

As an example of the performance of the different interatomic electrostatic terms, the following Tables include the corresponding data for the atomic contacts between two acetic acid monomers. The atom types there shown correspond to the GAFF classification.[10]

Figure S4: Atomic numbering of the acetic acid dimer. Oxygen atoms are in red, hydrogens in white and carbons in grey (Jmol image).

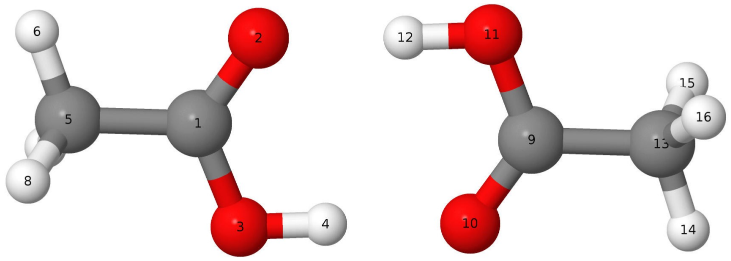

Table S4: Polar contacts in the E_{ele}^{IJ} and $E_{ele}^{0,IJ}$ interactions.

Atom-A	Atom-B	Atype-A	Atype-B	E_{ele}^{IJ}	$E_{ele}^{0,IJ}$	Difference
H -4	O -10	ho	o	-181.1	-147.8	33.3
O -2	H -12	o	ho	-181.1	-147.8	33.3
C -1	H -12	c	ho	190.9	165.4	25.4
H -4	C -9	ho	c	190.9	165.4	25.4
O -3	O -10	oh	o	237.7	214.0	23.7
O -2	O -11	o	oh	237.7	214.0	23.6
O -3	H -12	oh	ho	-122.4	-101.6	20.8
H -4	O -11	ho	oh	-122.4	-101.6	20.8
O -3	O -11	oh	oh	193.8	174.6	19.1
H -4	H -12	ho	ho	74.4	56.9	17.4
C -1	O -11	c	oh	-274.7	-258.4	16.2
O -3	C -9	oh	c	-274.6	-258.4	16.2
O -2	O -10	o	o	203.0	188.3	14.7
C -1	O -10	c	o	-274.4	-261.2	13.2
O -2	C -9	o	c	-274.4	-261.2	13.1
C -1	C -9	c	c	347.0	342.1	4.9

Table S5: Mixed contacts in the E_{ele}^{IJ} and $E_{ele}^{0,IJ}$ interactions.

Atom-A	Atom-B	Atype-A	Atype-B	E_{ele}^{IJ}	$E_{ele}^{0,IJ}$	Difference
H -7	C -9	hc	c	2.0	0.8	1.2
C -1	H -15	c	hc	2.0	0.9	1.2
C -1	H -16	c	hc	2.0	0.9	1.2
H -8	C -9	hc	c	2.0	0.9	1.1
C -1	C -13	c	c3	21.4	22.5	1.0
H -7	O -10	hc	o	-1.6	-0.6	0.9
O -2	H -15	o	hc	-1.6	-0.6	0.9
O -2	H -16	o	hc	-1.6	-0.6	0.9
H -8	O -10	hc	o	-1.6	-0.6	0.9
O -3	H -16	oh	hc	-1.7	-0.7	0.9
C -5	H -12	c3	ho	11.0	10.1	0.9
C -5	C -9	c3	c	21.5	22.4	0.9
O -3	H -15	oh	hc	-1.6	-0.7	0.9
H -7	O -11	hc	oh	-1.6	-0.7	0.9
H -8	O -11	hc	oh	-1.6	-0.7	0.9
H -4	C -13	ho	c3	11.0	10.1	0.9
H -6	C -9	hc	c	2.1	2.8	0.7
C -1	H -14	c	hc	2.1	2.8	0.7
H -4	H -15	ho	hc	1.1	0.5	0.6
H -4	H -16	ho	hc	1.1	0.5	0.6
H -7	H -12	hc	ho	1.1	0.5	0.6
H -8	H -12	hc	ho	1.1	0.5	0.6
H -6	O -11	hc	oh	-1.5	-2.0	0.5
O -3	H -14	oh	hc	-1.5	-2.0	0.5
O -2	H -14	o	hc	-1.7	-2.2	0.5
H -6	O -10	hc	o	-1.7	-2.2	0.5
O -2	C -13	o	c3	-16.8	-17.1	0.2
H -4	H -14	ho	hc	1.0	1.2	0.2
H -6	H -12	hc	ho	1.0	1.2	0.2
C -5	O -10	c3	o	-16.9	-17.0	0.2
C -5	O -11	c3	oh	-16.9	-16.8	0.1
O -3	C -13	oh	c3	-16.9	-16.9	0.0

Table S6: Non-polar contacts in the E_{ele}^{IJ} and $E_{ele}^{0,IJ}$ interactions.

Atom-A	Atom-B	Atype-A	Atype-B	E_{ele}^{IJ}	$E_{ele}^{0,IJ}$	Difference
C -5	C -13	c3	c3	1.4	1.6	0.2
C -5	H -15	c3	hc	0.1	0.1	0.1
C -5	H -16	c3	hc	0.1	0.1	0.1
H -7	C -13	hc	c3	0.1	0.1	0.1
H -8	C -13	hc	c3	0.1	0.1	0.1
C -5	H -14	c3	hc	0.1	0.2	0.1
H -6	C -13	hc	c3	0.1	0.2	0.1
H -6	H -14	hc	hc	0.0	0.0	0.0
H -7	H -15	hc	hc	0.0	0.0	0.0
H -7	H -16	hc	hc	0.0	0.0	0.0
H -8	H -15	hc	hc	0.0	0.0	0.0
H -8	H -16	hc	hc	0.0	0.0	0.0
H -6	H -15	hc	hc	0.0	0.0	0.0
H -6	H -16	hc	hc	0.0	0.0	0.0
H -7	H -14	hc	hc	0.0	0.0	0.0
H -8	H -14	hc	hc	0.0	0.0	0.0

Table S7: Polar contacts in the $E_{ele}^{0,IJ}$ and $E_{ele,mp}^{0,IJ}$ interactions.

Atom-A	Atom-B	Atype-A	Atype-B	$E_{ele}^{0,IJ}$	$E_{ele,mp}^{0,IJ}$	Difference
O -3	O -10	oh	o	214.0	219.9	5.9
O -2	O -11	o	oh	214.0	219.9	5.9
O -2	O -10	o	o	188.3	192.5	4.2
C -1	O -10	c	o	-261.2	-265.3	4.0
O -2	C -9	o	c	-261.2	-265.2	4.0
H -4	O -10	ho	o	-147.8	-151.5	3.7
O -2	H -12	o	ho	-147.8	-151.5	3.7
O -3	C -9	oh	c	-258.4	-260.3	1.8
C -1	O -11	c	oh	-258.4	-260.3	1.8
O -3	O -11	oh	oh	174.6	176.4	1.7
H -4	H -12	ho	ho	56.9	58.2	1.3
O -3	H -12	oh	ho	-101.6	-102.8	1.2
H -4	O -11	ho	oh	-101.6	-102.8	1.2
C -1	C -9	c	c	342.1	342.7	0.7
H -4	C -9	ho	c	165.4	165.2	0.2
C -1	H -12	c	ho	165.4	165.2	0.2

Table S8: Mixed contacts in the $E_{ele}^{0,IJ}$ and $E_{ele,mp}^{0,IJ}$ interactions.

Atom-A	Atom-B	Atype-A	Atype-B	$E_{ele}^{0,IJ}$	$E_{ele,mp}^{0,IJ}$	Difference
C -1	C -13	c	c3	22.5	22.2	0.3
C -5	C -9	c3	c	22.4	22.2	0.3
C -5	H -12	c3	ho	10.1	9.9	0.2
H -4	C -13	ho	c3	10.1	9.9	0.2
O -3	C -13	oh	c3	-16.9	-16.7	0.1
C -5	O -11	c3	oh	-16.8	-16.7	0.1
O -2	C -13	o	c3	-17.1	-17.0	0.0
C -5	O -10	c3	o	-17.0	-17.0	0.0
H -6	O -10	hc	o	-2.2	-2.2	0.0
O -2	H -14	o	hc	-2.2	-2.2	0.0
H -8	C -9	hc	c	0.9	0.9	0.0
H -8	H -12	hc	ho	0.5	0.5	0.0
C -1	H -16	c	hc	0.9	0.8	0.0
H -4	H -14	ho	hc	1.2	1.2	0.0
H -7	C -9	hc	c	0.8	0.8	0.0
C -1	H -15	c	hc	0.9	0.8	0.0
H -7	H -12	hc	ho	0.5	0.5	0.0
H -4	H -15	ho	hc	0.5	0.5	0.0
H -4	H -16	ho	hc	0.5	0.5	0.0
H -6	H -12	hc	ho	1.2	1.2	0.0
H -7	O -11	hc	oh	-0.7	-0.7	0.0
C -1	H -14	c	hc	2.8	2.8	0.0
H -6	C -9	hc	c	2.8	2.8	0.0
H -8	O -10	hc	o	-0.6	-0.6	0.0
H -6	O -11	hc	oh	-2.0	-2.0	0.0
O -2	H -16	o	hc	-0.6	-0.6	0.0
O -3	H -15	oh	hc	-0.7	-0.7	0.0
O -3	H -16	oh	hc	-0.7	-0.7	0.0
H -8	O -11	hc	oh	-0.7	-0.7	0.0
O -3	H -14	oh	hc	-2.0	-2.0	0.0
O -2	H -15	o	hc	-0.6	-0.6	0.0
H -7	O -10	hc	o	-0.6	-0.6	0.0

Table S9: Non-polar contacts in the $E_{ele}^{0,IJ}$ and $E_{ele,mp}^{0,IJ}$ interactions.

Atom-A	Atom-B	Atype-A	Atype-B	$E_{ele}^{0,IJ}$	$E_{ele,mp}^{0,IJ}$	Difference
C -5	C -13	c3	c3	1.6	1.5	0.0
H -8	C -13	hc	c3	0.1	0.1	0.0
H -7	H -15	hc	hc	0.0	0.0	0.0
H -8	H -16	hc	hc	0.0	0.0	0.0
H -6	H -15	hc	hc	0.0	0.0	0.0
H -6	H -16	hc	hc	0.0	0.0	0.0
H -7	H -14	hc	hc	0.0	0.0	0.0
H -6	H -14	hc	hc	0.0	0.0	0.0
H -8	H -14	hc	hc	0.0	0.0	0.0
C -5	H -14	c3	hc	0.2	0.2	0.0
H -6	C -13	hc	c3	0.2	0.2	0.0
C -5	H -15	c3	hc	0.1	0.1	0.0
C -5	H -16	c3	hc	0.1	0.1	0.0
H -7	C -13	hc	c3	0.1	0.1	0.0
H -7	H -16	hc	hc	0.0	0.0	0.0
H -8	H -15	hc	hc	0.0	0.0	0.0

Table S10: Polar contacts in the $E_{ele}^{0,IJ}$ and $E_{ele,AMOEBA}^{IJ}$ interactions.

Atom-A	Atom-B	Atype-A	Atype-B	$E_{ele}^{0,IJ}$	$E_{ele,AMOEBA}^{IJ}$	Difference
C -1	C -9	c	c	342.1	63.6	278.5
C -1	O -11	c	oh	-258.4	-32.7	225.7
O -3	C -9	oh	c	-258.4	-32.7	225.7
O -2	C -9	o	c	-261.2	-55.5	205.7
C -1	O -10	c	o	-261.2	-55.5	205.7
O -2	O -11	o	oh	214.0	30.7	183.3
O -3	O -10	oh	o	214.0	30.7	183.3
O -3	O -11	oh	oh	174.6	15.7	158.9
O -2	O -10	o	o	188.3	43.8	144.5
C -1	H -12	c	ho	165.4	27.4	138.0
H -4	C -9	ho	c	165.4	27.4	138.0
O -2	H -12	o	ho	-147.8	-34.0	113.8
H -4	O -10	ho	o	-147.8	-34.0	113.8
H -4	O -11	ho	oh	-101.6	-11.2	90.4
O -3	H -12	oh	ho	-101.6	-11.2	90.4
H -4	H -12	ho	ho	56.9	7.6	49.3

Table S11: Mixed contacts in the $E^{0,IJ}_{ele}$ and $E^{IJ}_{ele,AMOEBA}$ interactions.

Atom-A	Atom-B	Atype-A	Atype-B	$E^{0,IJ}_{ele}$	$E^{IJ}_{ele,AMOEBA}$	Difference
C -1	C -13	c	c3	22.5	-11.7	34.2
C -5	C -9	c3	c	22.4	-11.7	34.1
O -2	C -13	o	c3	-17.1	9.7	26.8
C -5	O -10	c3	o	-17.0	9.7	26.8
O -3	C -13	oh	c3	-16.9	6.3	23.2
C -5	O -11	c3	oh	-16.8	6.3	23.1
H -4	C -13	ho	c3	10.1	-4.4	14.6
C -5	H -12	c3	ho	10.1	-4.4	14.5
H -7	C -9	hc	c	0.8	3.9	3.1
C -1	H -15	c	hc	0.9	3.9	3.1
C -1	H -16	c	hc	0.9	3.9	3.1
H -8	C -9	hc	c	0.9	3.9	3.1
H -7	O -10	hc	o	-0.6	-3.4	2.8
O -2	H -15	o	hc	-0.6	-3.4	2.7
O -2	H -16	o	hc	-0.6	-3.3	2.7
H -8	O -10	hc	o	-0.6	-3.3	2.7
O -3	H -16	oh	hc	-0.7	-2.1	1.3
O -3	H -15	oh	hc	-0.7	-2.1	1.3
H -7	O -11	hc	oh	-0.7	-2.1	1.3
H -8	O -11	hc	oh	-0.7	-2.1	1.3
C -1	H -14	c	hc	2.8	3.9	1.1
H -6	C -9	hc	c	2.8	3.9	1.1
O -2	H -14	o	hc	-2.2	-3.1	0.9
H -6	O -10	hc	o	-2.2	-3.1	0.9
H -4	H -16	ho	hc	0.5	1.3	0.8
H -4	H -15	ho	hc	0.5	1.3	0.8
H -7	H -12	hc	ho	0.5	1.3	0.8
H -8	H -12	hc	ho	0.5	1.3	0.8
H -4	H -14	ho	hc	1.2	1.6	0.4
H -6	H -12	hc	ho	1.2	1.6	0.4
O -3	H -14	oh	hc	-2.0	-2.3	0.2
H -6	O -11	hc	oh	-2.0	-2.3	0.2

Table S12: Non-polar contacts in the $E_{ele}^{0,IJ}$ and $E_{ele,AMOEBA}^{IJ}$ interactions.

Atom-A	Atom-B	Atype-A	Atype-B	$E_{ele}^{0,IJ}$	$E_{ele,AMOEBA}^{IJ}$	Difference
C -5	H -14	c3	hc	0.2	-0.8	1.0
H -6	C -13	hc	c3	0.2	-0.8	1.0
H -8	C -13	hc	c3	0.1	-0.9	0.9
C -5	H -16	c3	hc	0.1	-0.9	0.9
C -5	H -15	c3	hc	0.1	-0.8	0.9
H -7	C -13	hc	c3	0.1	-0.8	0.9
C -5	C -13	c3	c3	1.6	2.5	0.9
H -8	H -16	hc	hc	0.0	0.3	0.3
H -7	H -15	hc	hc	0.0	0.3	0.3
H -6	H -15	hc	hc	0.0	0.3	0.3
H -7	H -14	hc	hc	0.0	0.3	0.3
H -8	H -14	hc	hc	0.0	0.3	0.3
H -6	H -16	hc	hc	0.0	0.3	0.3
H -8	H -15	hc	hc	0.0	0.3	0.3
H -7	H -16	hc	hc	0.0	0.3	0.3
H -6	H -14	hc	hc	0.0	0.3	0.3

Table S13: Polar contacts in the $E_{ele}^{0,IJ}$ and $E_{ele,RESP}^{IJ}$ interactions.

Atom-A	Atom-B	Atype-A	Atype-B	$E_{ele}^{0,IJ}$	$E_{ele,GAFF}^{IJ}$	Difference
C -1	C -9	c	c	342.1	54.4	287.7
O -2	C -9	o	c	-261.2	-48.1	213.1
C -1	O -10	c	o	-261.2	-48.3	213.0
O -3	C -9	oh	c	-258.4	-47.3	211.2
C -1	O -11	c	oh	-258.4	-47.4	211.0
O -2	O -11	o	oh	214.0	48.4	165.6
O -3	O -10	oh	o	214.0	48.5	165.5
O -2	O -10	o	o	188.3	38.2	150.1
O -3	O -11	oh	oh	174.6	36.2	138.4
H -4	C -9	ho	c	165.4	45.5	119.9
C -1	H -12	c	ho	165.4	45.7	119.8
O -2	H -12	o	ho	-147.8	-54.4	93.4
H -4	O -10	ho	o	-147.8	-54.4	93.4
H -4	O -11	ho	oh	-101.6	-32.0	69.7
O -3	H -12	oh	ho	-101.6	-32.0	69.6
H -4	H -12	ho	ho	56.9	27.8	29.2

Table S14: Mixed contacts in the $E_{ele}^{0,IJ}$ and $E_{ele,RESP}^{IJ}$ interactions.

Atom-A	Atom-B	Atype-A	Atype-B	$E_{ele}^{0,IJ}$	$E_{ele,GAFF}^{IJ}$	Difference
C -5	C -9	c3	c	22.4	-14.0	36.4
C -1	C -13	c	c3	22.5	-13.8	36.2
C -5	O -10	c3	o	-17.0	12.1	29.1
O -2	C -13	o	c3	-17.1	11.8	28.9
C -5	O -11	c3	oh	-16.8	12.1	28.9
O -3	C -13	oh	c3	-16.9	11.8	28.7
C -5	H -12	c3	ho	10.1	-10.6	20.7
H -4	C -13	ho	c3	10.1	-10.4	20.5
H -7	C -9	hc	c	0.8	4.4	3.6
H -8	C -9	hc	c	0.9	4.4	3.5
C -1	H -15	c	hc	0.9	4.4	3.5
C -1	H -16	c	hc	0.9	4.4	3.5
H -7	O -10	hc	o	-0.6	-3.9	3.3
H -8	O -10	hc	o	-0.6	-3.9	3.2
O -2	H -15	o	hc	-0.6	-3.8	3.2
O -2	H -16	o	hc	-0.6	-3.8	3.2
H -7	O -11	hc	oh	-0.7	-3.7	3.0
H -8	O -11	hc	oh	-0.7	-3.7	3.0
O -3	H -15	oh	hc	-0.7	-3.6	2.9
O -3	H -16	oh	hc	-0.7	-3.6	2.9
H -7	H -12	hc	ho	0.5	3.1	2.6
H -8	H -12	hc	ho	0.5	3.1	2.6
H -4	H -15	ho	hc	0.5	3.1	2.6
H -4	H -16	ho	hc	0.5	3.1	2.6
H -6	H -12	hc	ho	1.2	3.4	2.2
H -4	H -14	ho	hc	1.2	3.4	2.2
H -6	O -11	hc	oh	-2.0	-3.9	1.9
O -3	H -14	oh	hc	-2.0	-3.9	1.9
H -6	C -9	hc	c	2.8	4.4	1.6
C -1	H -14	c	hc	2.8	4.3	1.6
H -6	O -10	hc	o	-2.2	-3.6	1.4
O -2	H -14	o	hc	-2.2	-3.6	1.4

Table S15: Non-polar contacts in the $E_{ele}^{0,IJ}$ and $E_{ele,RESP}^{IJ}$ interactions.

Atom-A	Atom-B	Atype-A	Atype-B	$E_{ele}^{0,IJ}$	$E_{ele,GAFF}^{IJ}$	Difference
C -5	C -13	c3	c3	1.6	3.9	2.3
C -5	H -14	c3	hc	0.2	-1.2	1.4
H -6	C -13	hc	c3	0.2	-1.2	1.4
C -5	H -15	c3	hc	0.1	-1.2	1.3
C -5	H -16	c3	hc	0.1	-1.2	1.3
H -8	C -13	hc	c3	0.1	-1.2	1.3
H -7	C -13	hc	c3	0.1	-1.2	1.3
H -8	H -16	hc	hc	0.0	0.4	0.4
H -7	H -15	hc	hc	0.0	0.4	0.4
H -6	H -15	hc	hc	0.0	0.4	0.4
H -6	H -16	hc	hc	0.0	0.4	0.4
H -7	H -14	hc	hc	0.0	0.4	0.4
H -7	H -16	hc	hc	0.0	0.4	0.4
H -8	H -14	hc	hc	0.0	0.4	0.4
H -8	H -15	hc	hc	0.0	0.4	0.4
H -6	H -14	hc	hc	0.0	0.4	0.4

References

(1) Pendás, A. M.; Blanco, M. A.; Francisco, E. Two-electron integrations in the quantum theory of atoms in molecules. *J. Chem. Phys.* **2004**, *120*, 4581–4592.

(2) Bader, R. *Atoms in molecules : a quantum theory*; Clarendon Press: Oxford New York, 1990.

(3) Popelier, P. L. A.; Kosov, D. S. Atom–atom partitioning of intramolecular and intermolecular Coulomb energy. *J. Chem. Phys.* **2001**, *114*, 6539–6547.

(4) Rafat, M.; Popelier, P. L. A. A convergent multipole expansion for 1, 3 and 1, 4 Coulomb interactions. *J. Chem. Phys.* **2006**, *124*, 144102.

(5) Stone, A. *The Theory of Intermolecular Forces*; Oxford University Press, 2013.

(6) Freitag, M. A.; Gordon, M. S.; Jensen, J. H.; Stevens, W. J. Evaluation of charge

penetration between distributed multipolar expansions. *J. Chem. Phys.* **2000**, *112*, 7300–7306.

(7) Rackers, J. A.; Wang, Q.; Liu, C.; Piquemal, J.-P.; Ren, P.; Ponder, J. W. An optimized charge penetration model for use with the AMOEBA force field. *Phys. Chem. Chem. Phys.* **2017**, *19*, 276–291.

(8) Bojarowski, S. A.; Kumar, P.; Dominiak, P. M. A Universal and Straightforward Approach to Include Penetration Effects in Electrostatic Interaction Energy Estimation. *ChemPhysChem* **2016**, *17*, 2455–2460.

(9) Wang, B.; Truhlar, D. G. Including Charge Penetration Effects in Molecular Modeling. *J. Chem. Theory Comput.* **2010**, *6*, 3330–3342.

(10) Wang, J.; Wolf, R. M.; Caldwell, J. W.; Kollman, P. A.; Case, D. A. Development and testing of a general amber force field. *J. Comput. Chem.* **2004**, *25*, 1157–1174.
